# Characterization and commissioning of a new collaborative multi-modality radiotherapy platform

**DOI:** 10.1007/s13246-023-01255-2

**Published:** 2023-06-28

**Authors:** Zhongfei Wang, Xiaohuan Sun, Wei Wang, Te Zhang, Liting Chen, Jie Duan, Siqi Feng, Yinzhu Chen, Zhiwei Wei, Jian Zang, Feng Xiao, Lina Zhao

**Affiliations:** 1 Department of Radiation Oncology, Xijing Hospital, Fourth Military Medical University, 710032 Xi’an, Shaanxi Province P.R. China; 2 Our United Corporation, 710018 Xi’an, Shaanxi Province P.R. China

**Keywords:** Multi-modality radiotherapy, Acceptance testing, Commissioning, Dosimetry validation

## Abstract

TaiChi, a new multi-modality radiotherapy platform that integrates a linear accelerator, a focusing gamma system, and a kV imaging system within an enclosed O-ring gantry, was introduced into clinical application. This work aims to assess the technological characteristics and commissioning results of the TaiChi platform. The acceptance testing and commissioning were performed following the manufacturer’s customer acceptance tests (CAT) and several AAPM Task Group (TG) reports/guidelines. Regarding the linear accelerator (linac), all applicable validation measurements recommended by the MPPG 5.a (basic photon beam model validation, intensity-modulated radiotherapy (IMRT)/volumetric-modulated arc therapy (VMAT) validation, end-to-end(E2E) tests, and patient-specific quality assurance (QA)) were performed. For the focusing gamma system, the absorbed doses were measured using a PTW31014 ion chamber (IC) and PTW60016 diode detector. EBT3 films and a PTW60016 diode detector were employed to measure the relative output factors (ROFs). The E2E tests were performed using PTW31014 IC and EBT3 films. The coincidences between the imaging isocenter and the linac/gamma mechanical isocenter were investigated using EBT3 films. The image quality was evaluated regarding the contrast-to-noise ratio (CNR), spatial resolution, and uniformity. All tests included in the CAT met the manufacturer’s specifications. All MPPG 5.a measurements complied with the tolerances. The confidence limits for IMRT/VMAT point dose and dose distribution measurements were achieved according to TG-119. The point dose differences were below 1.68% and gamma passing rates (3%/2 mm) were above 95.1% for the linac E2E tests. All plans of patient-specific QA had point dose differences below 1.79% and gamma passing rates above 96.1% using the 3%/2 mm criterion suggested by TG-218. For the focusing gamma system, the differences between the calculated and measured absorbed doses were below 1.86%. The ROFs calculated by the TPS were independently confirmed within 2% using EBT3 films and a PTW60016 detector. The point dose differences were below 2.57% and gamma passing rates were above 95.3% using the 2%/1 mm criterion for the E2E tests. The coincidences between the imaging isocenter and the linac/gamma mechanical isocenter were within 0.5 mm. The image quality parameters fully complied with the manufacturer’s specifications regarding the CNR, spatial resolution, and uniformity. The multi-modality radiotherapy platform complies with the CAT and AAPM commissioning criteria. The commissioning results demonstrate that this platform performs well in mechanical and dosimetry accuracy.

**Supplementary Information:**

The online version contains supplementary material available at 10.1007/s13246-023-01255-2.

## Introduction

The linear accelerator (linac) is the dominant machine in modern radiotherapy [1]. It is efficient for treating large tumors with unclear boundaries with different delivery techniques, including three-dimensional conformal radiation therapy (3D-CRT), intensity-modulated radiation therapy (IMRT), and volumetric-modulated arc therapy (VMAT). The Gamma Knife represents the gold standard for intracranial stereotactic radiosurgery [2, 3], using dedicated equipment allowing for the treatment of tumoral or non-tumoral disorders, as well as functional interventions for small brain lesions with very high precision. With the improvement in accuracy of linacs [4, 5] and the development of image-guided technology [6, 7], linac-based radiosurgery has been developed and clinically implemented. Many studies have been conducted comparing the two modalities in treating tumors within the brain. Compared to linac-based radiosurgery, the Gamma Knife has an advantage with better dose conformity and lower normal brain dose [8–11]. Thus, the linac and Gamma Knife are irreplaceable and complementary to a certain extent and are the primary means in precise tele-radiotherapy. Recently, TaiChi, a newly designed multi-modality form of radiotherapy, was introduced into the market by OUR United Corp (Xi’an, China). It integrates a linear accelerator, a focusing gamma system, and a kV imaging system within an enclosed O-ring gantry, providing collaborative multi-modality radiotherapy. The combination of the linac and focusing gamma system can realize X-ray intensity-modulated radiotherapy and γ-ray stereotactic radiotherapy on the same platform.

In 2021, the TaiChi platform was installed in our department and prepared for clinical usage. The aims of this report are (a) to introduce the technological characteristics and clinical workflow of this new treatment platform, and (b) to summarize the procedures and present the results of commissioning tests. The commissioning was performed mainly based on several American Association of Physicists in Medicine (AAPM) Task Group (TG) reports and guidelines for machine acceptance testing and commissioning, including quality assurance (QA) of medical accelerators (TG 142 [12]), commissioning and QA of treatment planning (MPPG 5.a [13]), IMRT commissioning (TG 119 [14]), tolerances and methodologies of IMRT QA (TG 218 [15]), calibration, dosimetry, and QA for gamma stereotactic radiosurgery (TG 178 [16]), and QA of CT-based IGRT systems (TG 179 [17]).

## Background

**Fig. 1:**
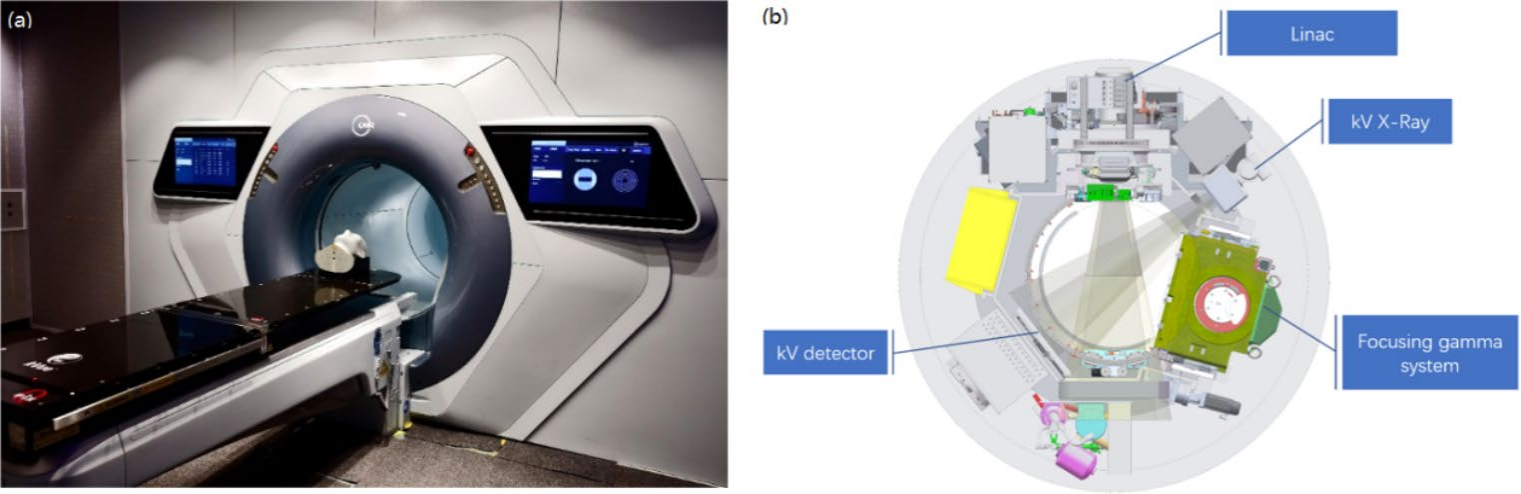
Schematic views of the TAICHI platform. **a** Diagram of the TaiChi platform. **b** Assembly drawing of the TaiChi platform. Linac, focusing gamma system, and kV imaging system (X-Ray and kV detector) are installed on an O ring gantry and share the same isocenter

The collaborative multi-modality radiotherapy platform combines a linac, a focusing gamma system, and a kV imaging system on an O-ring gantry, all of which share the same isocenter for both imaging and treatment. Figure 1 shows schematic views of the multi-modality radiotherapy platform, and Table 1 lists the main features of the platform. The gantry can rotate continuously clockwise and counterclockwise, which is achieved by using a slip ring and an on-gantry water cooling system. The slip ring serves as an electrical and signal connection for the stationary and rotating parts. It has two treatment heads, a linac head with a multileaf collimator (MLC) and a focusing gamma head. The linac can deliver a 6 MV flattening-filter-free (FFF) photon beam with a continuously variable dose rate of 50 to 1400 MU/min. The available delivery techniques include 3D-CRT, IMRT, and VMAT. Electron beams and wedges are not available on this platform. The linac has two pairs of collimating jaws, and the jaws transmission factor is 0.48%.The X jaws are designed to reduce photon leakage through the MLC leaf gaps when the MLC leaves move to the opposing side. The Y jaws and MLC define the radiation fields, and the field sizes range from 0.5 × 0.5 cm^2^ to 40 × 40 cm^2^. The MLC has 60 pairs of leaves, which includes 40 pairs of inner leaves with a 0.5 cm width (at the isocenter plane) and 20 pairs of outer leaves with a 1.0 cm width (at the isocenter plane). The MLC leaves have a maximum 4.0 cm/s leaf speed and a mean leaf leakage of 0.5%. The focusing gamma system consists of 18 Cobalt-60 sources, which are arranged to focus at the isocenter with different incident angles from − 13.3° to 13.3°. While keeping the isocenter unchanged, the gamma head can also swing 14° toward the patient’s head, giving the system more noncoplanar freedom to increase the dose gradient when treating small lesions. There are seven sets of available collimators. Among them, the Φ6 mm, Φ9 mm, Φ12 mm, Φ16 mm, and Φ20 mm collimators can be used for head and body treatment, while the Φ25 mm and Φ35 mm collimators are only used for body treatment. On the opposite side of the gantry ring, beam stoppers are designed for both treatment heads to reduce the shielding requirement for the treatment room. The imaging system consists of a kV x-ray source and a flat panel detector to provide 2D imaging and cone-beam computed tomography (CBCT). The main features are summarized in Table 2. Online kV imaging for patient position verification is performed using 2D x-rays or CBCT with relevant scanning protocols. A three-degrees-of-freedom (3DOF) treatment couch is used for patient transportation and image-guided automatic correction. It can travel a designated longitudinal distance of 860 mm between the external laser isocenter (also called the “virtual isocenter”) and the treatment isocenter inside the machine automatically when pressing the button “To Center” on the control panel. The bore opening is 96 cm in diameter, and the minimal distance between the gamma head and the isocenter is 37.5 cm.

**Table 1:** Main features of the TAICHI platform

Feature	Feature Description
Configuration	An O-ring gantry integrated with linac, focusing gamma system, and kV-imaging system
Gantry rotation	Continuous rotation
Treatment beam	Single-energy 6 MV (FFF) X-ray; Co-60 γ-ray
Nominal dose rate	X-ray:50-1400 MU/min (FFF); γ-ray: 530 cGy/min
MLC parameters	Single-layer opposed leaf banks Leaf width at isocenter (cm): 0.5 cm of 40 inner pairs, 1.0 cm of 20 outer pairs Leaf speed:0–4 cm/s Average leaf transmission (%):≤0.5%
Field size	Range from 0.5 × 0.5 cm^2^ to 40 × 40 cm^2^
Gamma system collimator	Φ6 mm, Φ9 mm, Φ12 mm, Φ16 mm, Φ25 mm and Φ35 mm
The γ-ray leakage at 1 m	≤ 1msv/h
Delivery techniques	3D-CRT, IMRT, VMAT
Treatment couch	3-DOF carbon fiber couch

**Table 2:** Description of the imaging system

Feature	Feature Description
Image Type	kV/kV(2D), kV CBCT(3D)
Detector	CsI
Dimension in pixels	1024 × 1024
thickness	1 mm
Scan FOV	Head:250 mm, range:170 mm Body:445 mm, range:190 mm
Image dose	2.5-8 mGy

Regarding the clinical image-guided radiation therapy (IGRT) workflow, the TaiChi platform has an external laser system to support patient positioning. First, the patient is positioned with external lasers to the “virtual” isocenter, then moved into the treatment isocenter automatically. Following a pretreatment kV-CBCT scan, image registration is performed based on the region of interest and bony landmarks. Then the treatment couch automatically moves to the target position and the treatment is delivered.

The TaiChi platform has its own oncology information system and treatment planning system (TPS) named RT PRO. The TPS beam modeling for linac is the same as other linacs. Beam data is collected during machine commissioning based on the requirement of the TPS. Source modeling for the focusing gamma system required beam data, tissue maximum ratio (TMR), percent depth dose (PDD), off-axis ratio (OAR), and output factors as the inputs. To reduce the workload of commissioning, the manufacturer offers a preconfigured beam model called “golden beam data” in the TPS, similar to the Halcyon [18] and TomoTherapy system [19]. The TPS supports treatment planning for the linac/MLC head and the focusing gamma head, as well as hybrid treatment planning, which means optimizing linac planning based on an existing focusing gamma plan.

## Methodology

The validations in this manuscript were performed in three parts: the linac, the focusing gamma system, and the kV imaging system.The acceptance testing items are listed in Table. S1 in the Supplementary Materials.

## The linac

### Acceptance testing

The acceptance testing was performed following the manufacturer’s customer acceptance tests (CAT). A conventional gantry and collimator spoke shot was performed with EBT3 films (Ashland Inc. Wayne, NJ, USA) to investigate the central axis beam variation due to gantry and collimator rotation [20], and the isocenter sphere diameter should be within 1 mm. The beam energy and beam symmetry were measured in the Blue Phantom^2^ (IBA, Schwarzenbruck, Germany) with a 90 cm source-surface distance (SSD). The relative dose at a depth of 10 cm should be (64.0 ± 2)% with the maximum dose of the percent depth dose (PDD) curve normalized to 100%, and the depth of the maximum dose (d_max_) should be (1.5 ± 0.2) mm. The beam symmetry at a depth of 10 cm should be within 3% for 5 × 5 cm^2^, 10 × 10 cm^2^, and 35 × 35 cm^2^ open fields. The output constancy with dose rates (50 and 1400 MU/min) and gantry angles (0°,90°,180°, and 270°) should be within 1%, and the output linearity should be within 2%.

### Validation of the basic photon beam model

#### Validation of field output factors

Field output factors were measured using the Blue Phantom^2^ at a 10 cm depth with a 90 cm SSD. A CC13 ion chamber (IC) (IBA) detector was used for large fields (≥ 4 × 4 cm^2^), and a PFD(IBA) diode detector was used for small fields (< 4 × 4 cm^2^). The field output correction factors of the PFD were derived from TRS-483 [21]. The 4 × 4 cm^2^ field was measured using both detectors and served as an intermediate field, and the daisy chaining strategy [22] was used to determine the small field output factors. The output factors measured in the Blue Phantom^2^ were compared to the output factors calculated in a virtual phantom (50 × 50 × 50 cm^3^) in the TPS.

#### Validation of MLC model

The MLC model of the TPS needs three parameters: MLC transmission factor, tongue-and-groove width, and leaf-tip width [23]. The MLC transmission factor is the ratio between the beam intensity after it has passed through the MLC leaves to beam intensity before entering the MLC. Measurements were performed using a CC13 IC in the Blue Phantom^2^ at a 1.5 cm depth with a 90 cm SSD. The tongue-and-groove width is the width of a region in cm (at the isocenter plane) that extends on the leaf side where the transmission equals the square root of the full leaf transmission. An abutment plan using the MLC leaf side was created and delivered, and the curves were measured using a PFD in the Blue Phantom^2^ at a 10 cm depth with a 90 cm SSD. The leaf-tip width is the width of a region in cm (at the isocenter plane) of the MLC leaf from the leaf tip to a point where the transmission equals the square root of the full leaf transmission. An abutment plan using the MLC leaf tip was created and delivered, and the curves were measured using a PFD in the Blue Phantom^2^ at a 10 cm depth with a 90 cm SSD. The values of three parameters which provided a good match between the measurements and calculations were selected as the MLC model parameters.

The picket Fence test was conducted to measure the position accuracy of the MLC leaf according to TG-82 [24]. Treatment plans of nine strip fields with 1 mm width and 20 mm gaps between each other were generated for four cardinal gantry angles on the TPS and delivered to EBT3 films embedded in a solid water phantom with a 100 cm source-axis distance (SAD). The exposed films were scanned with a V850Pro (Epson) scanner and analyzed with DoseLab film analysis software (Mobius Medical Systems, Houston, USA) to investigate the accuracy of the leaf positioning.

A dynamic IMRT field for the leaf speed test was created with a 5 mm MLC-defined gap moving from − 8 cm to + 8 cm in the leaf moving direction at a constant speed. The planned monitor unit was set to 149.3 MU to achieve the maximum MLC leaf speed (2.5 cm/s) at the maximum dose rate of 1400 MU/min. To delivery this field with different dose rates resulted in different leaf travel speeds. The IMRT filed was delivered with dose rate of 200, 500, 1000, and 1400 MU/min, respectively, and the corresponding leaf nominal travel speeds were 0.36, 0.89, 1.79, and 2.5 cm/s. The delivered dose distributions were measured with the Matrixx (IBA), the delivery times were recorded with a stopwatch. The travel speeds were calculated according to the delivery times and gap moving distance (16 cm). The gamma passing rates (2%/2 mm) of the dose distributions referencing to the result of 200 MU/min dose rate were analyzed with the myQA analysis software (IBA).

#### Validation of non-standard fields

The MPPG 5.a recommends a series of validation tests for non-standard fields which are different from the reference fields used for TPS modeling. The performed validation tests in this work are summarized in Table 3. Dose profiles and PDD curves were measured using a CC13 and PFD in the Blue phantom^2^. Dose profiles (inline and crossline) were measured at various depths (1.5 cm, 10 cm, and 25 cm). All the measurements were processed and analyzed with the OmniPro-Accept 7.4 software (IBA). Measured dose profiles and PDD curves were compared to the dose distributions calculated in a virtual phantom (50 × 50 × 50 cm^3^) in the TPS. The comparisons of the dose profiles and PDD curves were performed with a MATLAB program called Profile Comparison Tool [25]. A validation measurement was considered passing the MPPG 5.a criterion if the high-dose regions passed the 2%/2 mm criterion and both the low-dose tail and penumbra regions passed the 3%/3 mm criterion when the gamma passing rate was higher than 95%.

**Table 3:** Summary of the validation of MPPG 5.a tests. There are eight profiles in tests 5.3, 5.5, and 5.6 at deeper depths whose dose comparisons have gamma passing rates less than 95% with a 2%/2 mm criterion. When the 3%/3 mm criterion is applied, all the measurement profiles have gamma passing rates greater than 95%.Columns 3 and 4 list the number of measurements passing at 2%/2 mm and 3%/3 mm criterion

Test ID	Total measurements	Passed measurements (2%/2 mm)	Passed measurements (3%/3 mm)	Description
5.1	7	/	/	SSD = 90 cm. Point dose of axis and 10 cm off-axis at the depths of 5 cm,10 cm, and 20 cm. Field size:40 × 40 cm^2^
5.2	1	/	/	SSD = 100 cm. Point dose at a depth of 5 cm Field size:10 × 10 cm^2^
5.3	91	***87***	91	SSD = 90 cm. PDD and profiles (crossline and inline profile at the depths of 1.5 cm,10 cm, and 25 cm). Field size:1 × 1 cm^2^,2 × 2 cm^2^,3 × 3 cm^2^,4 × 4 cm^2^,5 × 5 cm^2^,6 × 6 cm^2^,8 × 8 cm^2^,10 × 10 cm^2^,15 × 15 cm^2^,20 × 20 cm^2^,30 × 30 cm^2^,35 × 35 cm^2^,40 × 40 cm^2^)
	49	49	49	SSD = 100 cm. PDD and profiles (crossline and inline profile at the depths of 1.5 cm,10 cm, and 25 cm). Field size: 2 × 2 cm^2^,10 × 10 cm^2^,25 × 25 cm^2^,2 × 10 cm^2^,10 × 2 cm^2^,2 × 25 cm^2^,25 × 2 cm^2^)
	21	21	21	SSD = 75 cm. PDD and profiles (crossline and inline profile at the depths of 1.5 cm,10 cm, and 25 cm). Field size:2 × 2 cm^2^,10 × 10 cm^2^,25 × 25 cm^2^)
5.4	7	7	7	SSD = 90 cm. PDD and profiles (crossline and inline profile at the depths of 1.5 cm,10 cm, and 25 cm)
5.5	7	***6***	7	SSD = 90 cm. PDD and profiles (crossline and inline profile at the depths of 1.5 cm,10 cm, and 25 cm)
5.6	7	***4***	7	SSD = 90 cm. PDD and profiles (crossline and inline profile at the depths of 1.5 cm,10 cm, and 25 cm)
5.7	7	7	7	SSD = 75 cm. PDD and profiles (crossline and inline profile at the depths of 1.5 cm,10 cm, and 25 cm)
7.1	7	7	7	SSD = 90 cm. PDD and profiles (crossline and inline profile at the depths of 1.5 cm,10 cm, and 25 cm)
7.2	2	/	/	SSD = 90 cm, point dose at a depth of 10 cm

### IMRT/VMAT dose validation

Dose validation of IMRT/VMAT was performed following the TG-119 report. TG-119 report provides a series of validation tests (including multi-target, prostate, head and neck(H&N), and C-shape cases) to evaluate the delivery accuracy of IMRT/VMAT [14]. A stack (30 × 30 × 15 cm^3^) of water-equivalent RW3 was used for point dose measurements. The point doses were measured using a CC13 IC with a DOSE1 (IBA) electrometer in both the high-dose region in the targets and the low-dose region in the avoidance structures. The plan dose distribution measurements were performed using ArcCHECK phantom (Sun Nuclear, Melbourne, FL) with a tighter criterion of 2%/2 mm and a 10% dose threshold (2%/2 mm/10%) during gamma analysis. The confidence limits of point dose measurements and dose distribution measurements were calculated using the formulation according to TG-119 report.

### End-to-end tests

An I’mRT Phantom (IBA) and a thorax phantom (CIRS, Norfolk, USA) were used for the E2E tests. A 2-arc VMAT was delivered to the I’mRT Phantom for a H&N case, a 2-arc VMAT was delivered to the CIRS thorax phantom for a lung SBRT case, and a 9-field IMRT was delivered to the I’mRT Phantom for a cervix case. Dose measurements were performed with a CC04 IC(IBA) and EBT3 films. Coronal film orientation was used in all tests. The exposed films were then scanned with a V850Pro (Epson) scanner and compared to the plan dose distributions using OmniPro I’mRT software (IBA) with a 3%/2 mm/10% criterion following the film scanning and calibration procedures [26]. The lung SBRT case can also be used for heterogeneity correction validation.

### Patient-specific QA

The patient-specific QA was performed using the ArcCHECK phantom for IMRT and VMAT treatment plans, including dose distribution measurements and point dose measurements with a CC04 IC inserted in the ArcCHECK phantom. The gamma analysis was evaluated using a 3%/2 mm/10% criterion as recommended by the TG-218 guideline [15], the gamma analysis using a tighter criterion of 2%/2 mm/10% was also performed.

## The focusing gamma system

### Point dose verification

The absorbed doses were measured at three locations in the manufacturer’s spherical phantom. The spherical phantom is made of polystyrene with a diameter of 160 mm, and there is an empty slot in the middle for inserting a film cassette or an ion chamber plate that keeps the ionization chamber in the expected position. In addition to the isocenter, point doses were measured at two other locations (3 cm anterior to isocenter and 5 cm posterior to isocenter). Three treatment plans for each collimator size were designed for the spherical phantom corresponding to the three measurement positions. A PTW31014 IC (PTW, Freiburg, Germany) was used for the Φ35 mm collimator, and a PTW60016 diode detector was used for all the collimators, then the PTW31014 IC and PTW60016 diode detector were calibrated using cross-calibration methods for the Φ35 mm collimator. The results were corrected by TRS-483 [21] when using the PTW60016 diode detector. The point dose differences between the measured doses and calculated doses were evaluated.

### ROF verification

Relative output factors of the seven collimators calculated by TPS were independently confirmed using the PTW60016 diode detector and EBT3 film measurements. Seven treatment plans for each collimator size were designed at the treatment isocenter for the spherical phantom. Relative output factors were relative with the Φ35 mm collimators. The results were corrected according to TRS-483 [21] when using the PTW60016 diode detector.

### End-to-end tests

**Fig. 2:**
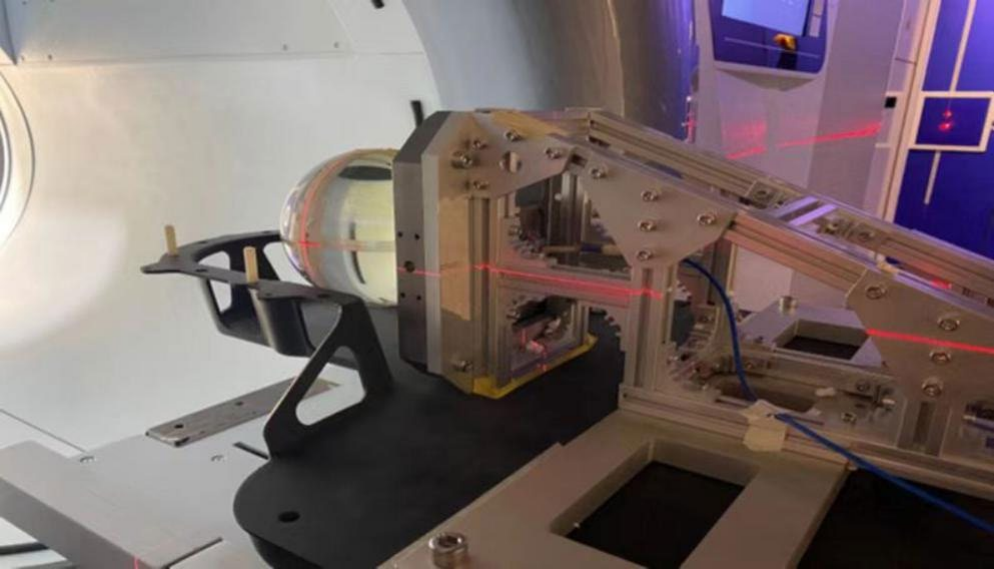
Photo of the manufacturer’s spherical phantom setup for the focusing gamma system E2E test (the brain case)

In order to test the dosimetry accuracy of plan delivery, two plans (brain and lung) were designed for the manufacturer’s spherical phantom and a thorax phantom (CIRS), respectively. The prescription doses of the plans were proportionally reduced, so that the film exposure would not exceed the saturation value. Figure 2 shows the setup of the spherical phantom for the E2E tests. Dose measurements were performed with a PTW31014 IC(PTW) and EBT3 films. Coronal film orientation was used in all tests. The point doses measured were compared to the point doses calculated by the TPS. The exposed films were scanned with a V850Pro (Epson) scanner and compared to the plan dose distributions using OmniPro I’mRT software (IBA) with a 2%/1mm/10% criterion.

## The kV imaging system

### Image quality

The image quality was evaluated regarding the CNR, spatial resolution, and uniformity. Phantom QCkVR-1(Sun Nuclear, USA) was used for kV image QA to investigate the CNR and spatial resolution, and CatPhan500 phantom (The Phantom Laboratory, USA) was used for kV-CBCT QA to investigate the CNR, spatial resolution and uniformity, respectively. The CNR of the 3D CBCT image was evaluated by four various contrast materials (Teflon, Acrylic, Air, and LDPE) of the CatPhan500 phantoms, and the CNR of the 2D kV image was evaluated by six various contrast materials of the QCkVR-1 phantoms. The spatial resolution was evaluated by counting the visually distinguishable line pairs on the acquired images of the two phantoms, and the uniformity was determined by the homogenous material in the CatPhan500 phantom.

### Geometric accuracy

**Fig. 3:**
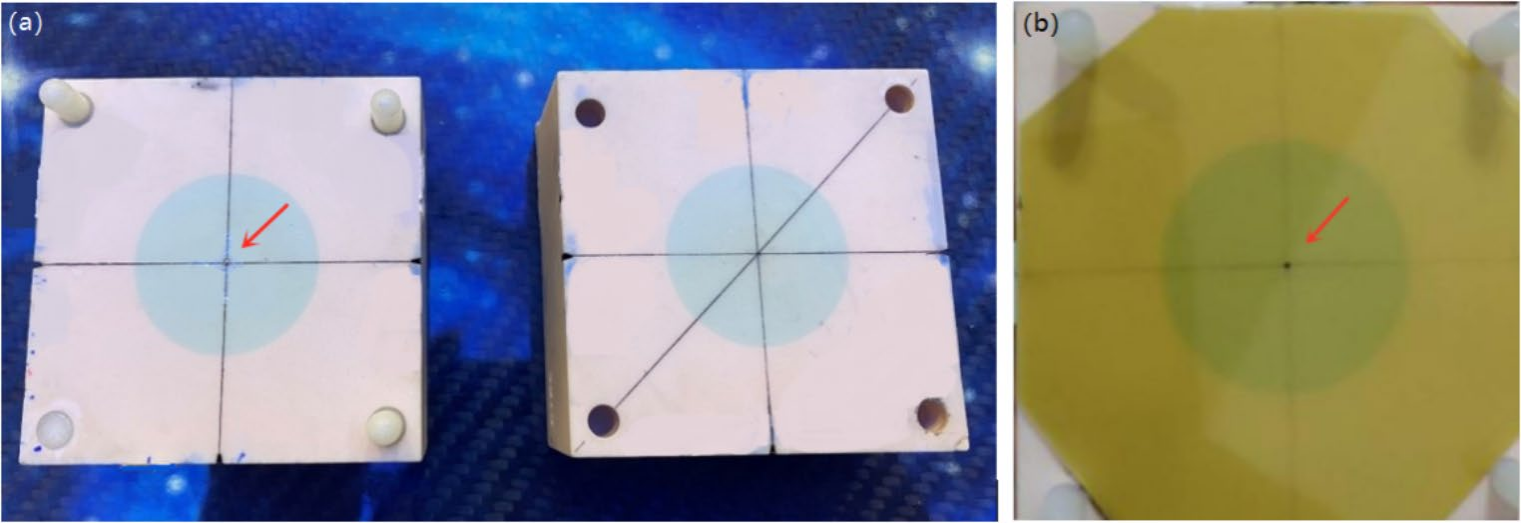
The measurement of imaging system geometric accuracy. **a** The film cube of CIRS038 phantom with a metal ballpoint at the center (indicated by the red arrow). **b** Mark the metal ballpoint on the film with a pen (indicated by the red arrow)

The coincidences between the imaging isocenter and the linac/gamma mechanical isocenter were evaluated using the CIRS038 phantom film cube with a metal ballpoint at the center and EBT3 films. The film was positioned in the phantom and marked with the ballpoint pen on the film, as shown in Fig. 3. Two orthogonal (coronal and sagittal) planes were measured in this session. The phantom was initially positioned with external lasers to the “virtual” isocenter, then moved into the mechanical isocenter automatically by moving the treatment couch. A CBCT of the film cube was acquired, and the metal ballpoint was moved to the image isocenter in the registration interface, then the offsets were corrected by moving the couch. The star shot plans were delivered with the linac, and gamma plans with a Φ6 mm collimator shot were delivered with the focusing gamma system. The exposed films were then scanned by V850Pro (Epson) and analyzed by PTW MEPHYSTO (PTW) to investigate the coincidences between the imaging isocenter and the linac/gamma mechanical isocenter.

## Results

### The linac

#### Acceptance testing

All verification tests included in the CAT were passed. The central axis beam variation due to the gantry and collimator rotation was within 0.5 mm (tolerance: <1 mm). The relative dose at the 10 cm depth was 64.3% and within the tolerance of 64.0 ± 2% with the maximum dose of PDD curve normalized to 100%. The measured dmax was 1.39 cm and within the tolerance of 1.5 ± 0.2 cm. The beam symmetry of crossline and inline profiles for the three open fields was below 1.2% and 1.15% (tolerance: <3%) respectively. The output constancy with dose rate was within 0.2% and the output constancy with gantry angle was within 0.5% (tolerance: <1%).The output linearity was below 0.9% (tolerance: <2%).

### Validation of the basic photon beam model

#### Validation of field output factors

**Fig. 4:**
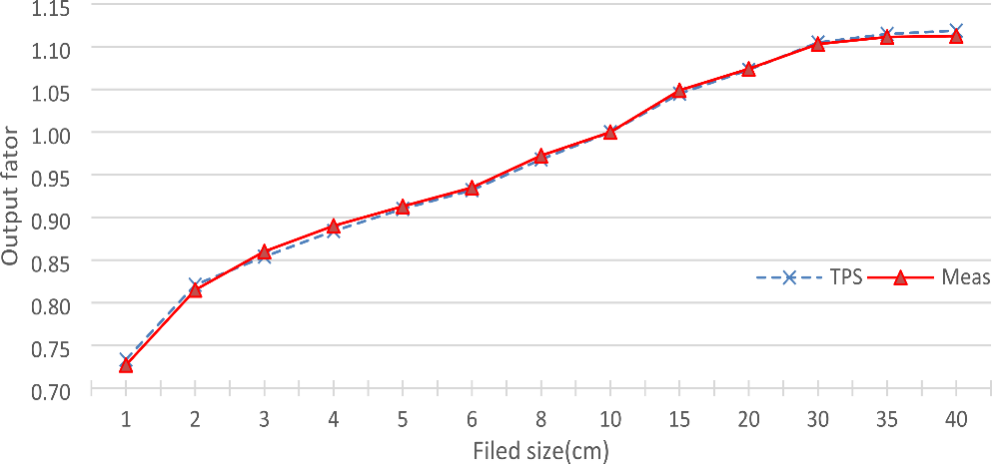
Output factors of the linac

Figure 4 displays calculated output factors by the TPS and the measured output factors in the Blue phantom^2^. The differences between the calculated and measured output factors were within 1% for all the fields.

#### Validation of MLC model

**Fig. 5:**
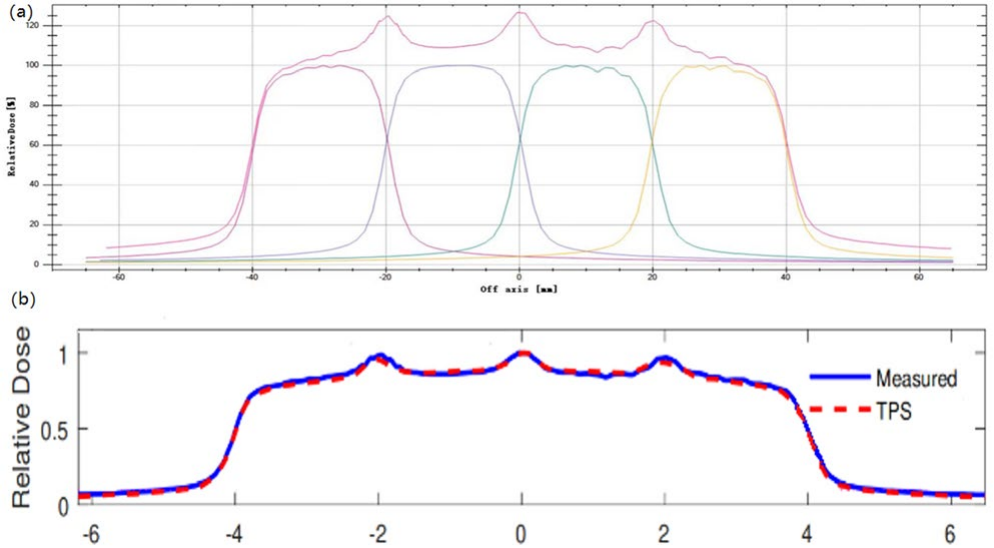
Validation of MLC leaf tip width. **a** The leaf tip width curves were measured by delivering the abutment plan in the Blue Phantom^2^. **b** The comparison of the leaf tip width curves between measured and calculated by the TPS. The value of the leaf tip width selected as the model parameter resulted in a good match between the measurement and calculation

The measured MLC transmission factor is 0.253% while the value in the TPS was 0.258%. The model parameter values of tongue-and-groove width and leaf-tip width were 0.1 cm and 0.02 cm, which provided a good dose-profile match. Figure 5 shows the validation result of the leaf-tip width. All MLC parameters also resulted in good agreement with IMRT and VMAT tests following TG-119.

The analysis of the picket fence test was done with Dose Lab software, and the maximum leaf position difference for four cardinal gantry angles was 0.44 mm. An example of the results is shown in Fig. S1 in the Supplementary Materials.

The measured leaf travel speeds were 0.35, 0.88, 1.78, and 2.52 cm/s for delivery with dose rates of 200, 500, 1000, and 1400 MU/min, respectively. The gamma passing rates (2%/2 mm) were 100% ,99.5% ,99.7% when comparing the dose distributions obtained at dose rates of 500, 1000, and 1400 MU/min with that of 200 MU/min. An example of the gamma analysis results is shown in Fig. S2 in the Supplementary Materials.

#### Validation of non-standard fields

**Fig. 6:**
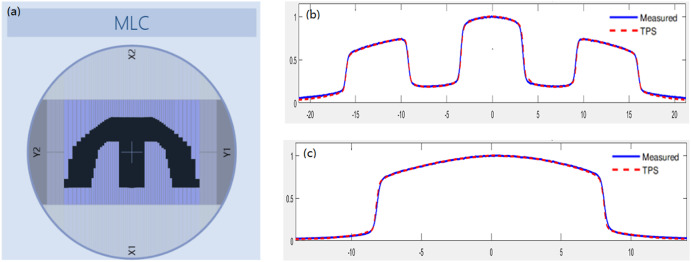
The test 5.5 of the MPPG 5.a. **a** Field apertures for test 5.5. **b** Inline profile. The poor agreement is mainly confined to the low-dose regions of the profile, which shows that the calculated doses in the TPS are lower than the measured doses. **c** Crossline profile

The validation tests and the results of MPPG 5.a are summarized in Table 3. All validation results met the MPPG 5.a tolerances. The dose differences in the planning and modeling module were below 0.12% in test (5.1) The dose difference in the test plan and reference calibration condition was below 0.3% in test (5.2) All PDD tests showed gamma passing rates greater than 95% with a criterion of 2%/2 mm. There were eight profiles in tests 5.3, 5.5 and 5.6 at deeper depths (≥ 25 cm) whose dose comparisons showed gamma passing rates less than 95% with a 2%/2 mm criterion. The disagreements were mainly confined to the low-dose regions of these profiles (the doses calculated by the TPS were lower than the doses measured in the Blue phantom^2^, as shown in Fig. 6). A criterion of 3%/3 mm was suggested by MPPG 5.a when evaluating penumbra and low-dose tail regions of a profile for the basic photon beam validation tests. When this criterion was applied, all validation test profiles showed gamma passing rates greater than 95%. Test 7.2 was implemented using two small fields (irregular, MLC-shaped fields), and the dose differences for test 7.2 were within 1.5%.

### IMRT/VMAT dose validation

The point dose and dose distribution validations of the TG-119 cases along with the confidence limits for IMRT/VMAT are summarized in Table 4. The point dose confidence limits were 3.55% (high-dose region: 3.40%, low-dose region: 4.01%) for IMRT and 3.95% (high-dose region: 3.67%, low-dose region: 3.87%) for VMAT. The confidence limits of point dose measurements suggested by TG-119 (tolerance: 4.5% for the high-dose region, 4.7% for the low-dose region) were achieved for both IMRT and VMAT. The dose distribution measurements had gamma passing rates above 95.5% and 95.4% for IMRT and VMAT using a criterion of 2%/2 mm which was tighter than 3%/3 mm used in TG-119. The confidence limits (2%/2 mm) for the dose distribution measurements were 4.95% for IMRT and 5.29% for VMAT, and both passed the suggested confidence limits for dose distribution measurements (tolerance:12.4% (3%/3 mm)).

**Table 4:** Summarizes the results of point dose measurements and dose distribution measurements for the TG-119 cases. Point dose confidence limit = |mean|+1.96σ, dose distribution confidence limit = (100-mean) + 1.96σ. Point dose difference: (measured dose -plan dose)/ prescription dose

Test	Location	Point dose difference (%)		Gamma passing rate (2%/2mm) (%)		
		IMRT	VMAT	IMRT		VMAT
MultiTarget	Central target	0.64	-0.44	95.5	98.7	
	Superior target	-0.23	-0.40			
	Inferior target	3.75	3.02			
Prostate	PTV	0.38	-0.15	98.5	99.6	
	Avoidance structure	-1.50	-2.47			
H&N	PTV	0.13	-0.95	98.6	97.5	
	Avoidance structure	-1.29	0.88			
C-shape (easy)	PTV	1.71	-1.45	97.7	95.4	
	Avoidance structure	-0.87	-1.69			
C-shape (hard)	PTV	-2.91	-3.27	97.4	97.6	
	Avoidance structure	0.92	0.58			
Confidence limit	High dose region	3.40	3.67	4.95	5.29	
	Low dose region	4.01	3.87			
	Overall combined	3.55	3.95			

### End-to-end tests

**Fig. 7:**
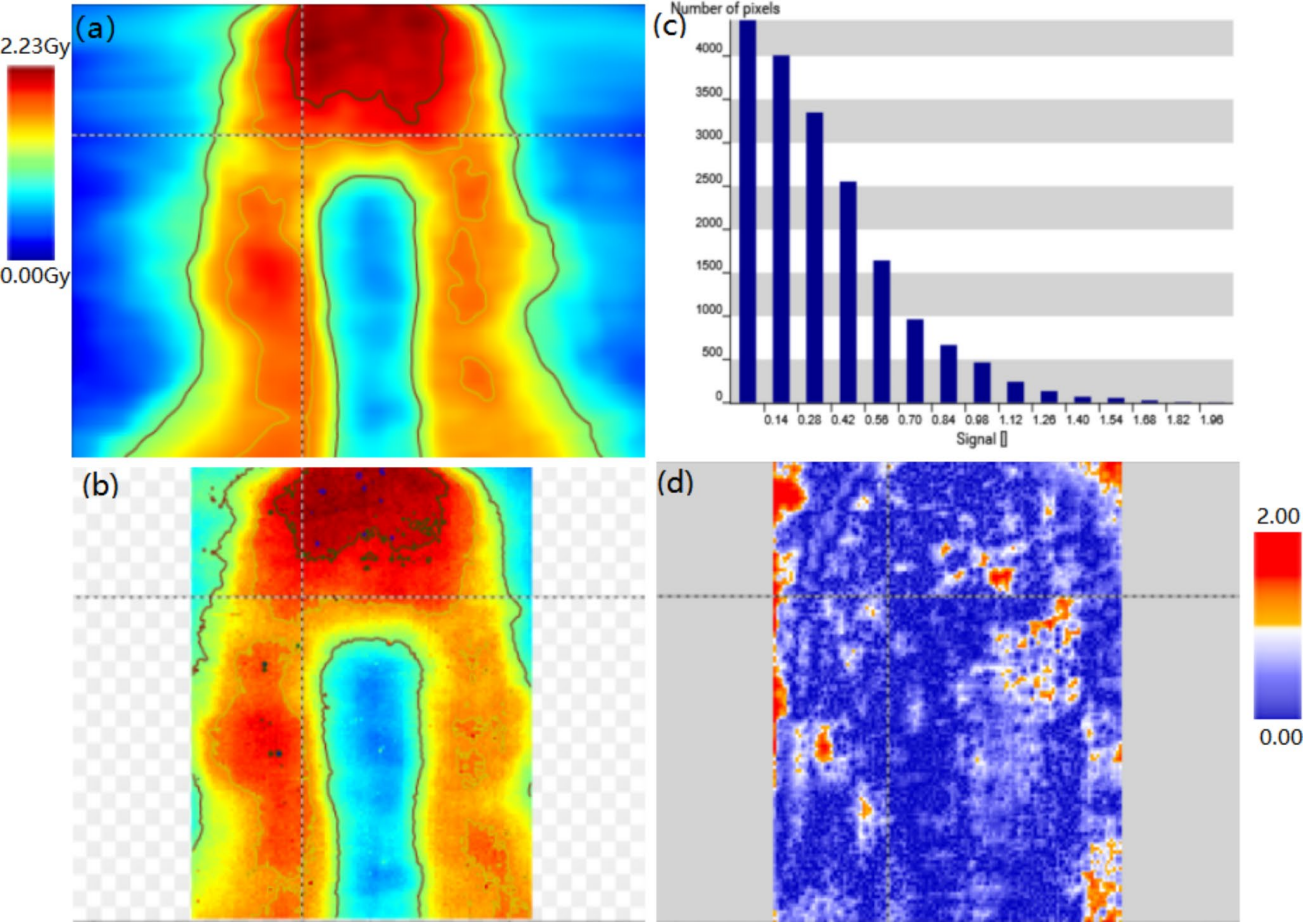
E2E test of the H&N VMAT case for linac. **a** Plan dose distribution. **b** Measured film dose distribution. **c** Histogram of gamma index. **d** Gamma index image with 3%/2mm (10% low dose threshold)

The point dose differences of the three E2E tests were − 1.58% (H&N), 1.01% (lung SBRT), and 1.68% (cervix), respectively. The EBT3 film measurements showed gamma passing rates (3%/2 mm /10%) of 95.1% (H&N), 96.2% (lung SBRT), and 95.3% (cervix), respectively. Figure 7 presents an example of the H&N VMAT case, and the gamma passing rate was 95.1% using a criterion of 3%/2 mm /10%.

### Patient-specific QA

The patient-specific QA results are given in Table 5. Patient-specific QA of IMRT/ VMAT showed gamma passing rates (2%/2 mm/10%) above 93.4% and 96.7%, and point dose differences below 1.55% and 1.80%, respectively. When analyzed using the 3%/2 mm criterion suggested by TG-218 [15], all patient-specific QA of IMRT/ VMAT had gamma passing rates above 96.1% and 99.1%, and the results were within the tolerance levels (point dose difference ≤ 2% and gamma passing rate ≥ 95%).

**Table 5:** The results of the patient-specific QA. Point dose difference: (measured dose-plan dose)/ plan dose

Site (case No.)	Delivery	Point dose difference (%)	Gamma passing rate (%)	
			(2%/2mm)	(3%/2mm)
H&N (2)	IMRT	1.55/0.74	99.1/95.3 100.0/99.5	99.7/97.5
	VMAT	0.13/1.74		100/99.9
Esophagus (2)	IMRT	0.80/0.99	93.90/98.0 96.9/99.5	96.3/98.5
	VMAT	-1.79/0.29		99.1/100
Lung (2)	IMRT	-0.25/0.11	97.3/98.2 100/99.7	99.5/99.3
	VMAT	-0.14/0.95		100/100
Stomach (2)	IMRT	-0.14/0.27	98.3/93.4	99.6/96.1
	VMAT	-0.81/0.07	99.10/98.3	99.8/99.5
Liver (1)	IMRT	0.51	99.0	99.2
	VMAT	-0.32	99.7	99.9
Cervix (2)	IMRT	-0.54/0.93	96.4/97.9 98.5/99.6	98.7/99.1
	VMAT	-0.68/1.80		99.8/99.8
Rectum (1)	IMRT	0.90	98.6 96.7	99.7
	VMAT	0.53		99.5
Confidence limit	IMRT	1.68	6.7	3.9
	VMAT	2.17	3.3	0.8

## The focusing gamma system

### Point dose verification

The absorbed dose verification results are listed in Table 6. The differences between the measured absorbed doses in the spherical phantom and the plan doses in the TPS ranged from − 0.94 to 1.86% at the isocenter, off-isocenter 30 and 50 mm of the spherical phantom, which met the requirement of ≤ 5%.

**Table 6:** The point dose difference between the measured and plan dose of the focusing gamma system. Point dose difference: (measured dose-plan dose)/ plan dose

Location	Point dose difference for each collimator(mm)						
	Φ6	Φ9	Φ12	Φ16	Φ20	Φ25	ΦΦ35
Isocenter	-0.77	-0.80%	-0.83%	-0.73%	-0.94%	-0.93%	-0.6%
3 cm anterior to isocenter	1.37%	1.40%	1.49%	1.53%	1.29%	1.38%	1.46%
5 cm posterior to isocenter	0.64%	1.78%	1.85%	1.86%	1.58%	1.44%	1.50%

### ROF verification

**Fig. 8:**
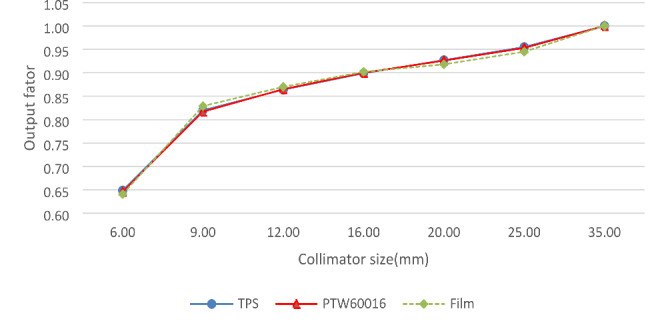
Output factors of the focusing gamma system

Figure 8 shows the ROFs measured by the PTW 60,016 diode detector and the EBT3 films and the ROFs calculated by the TPS. The maximum difference between the ROFs measured by PTW 60,016 diode detector and ROFs calculated by the TPS was − 0.46%. And the maximum difference between the ROFs measured by PTW 60,016 diode detector and ROFs calculated by the TPS was − 1.23% (for the Φ6 mm collimator).

### End-to-end tests

**Fig. 9:**
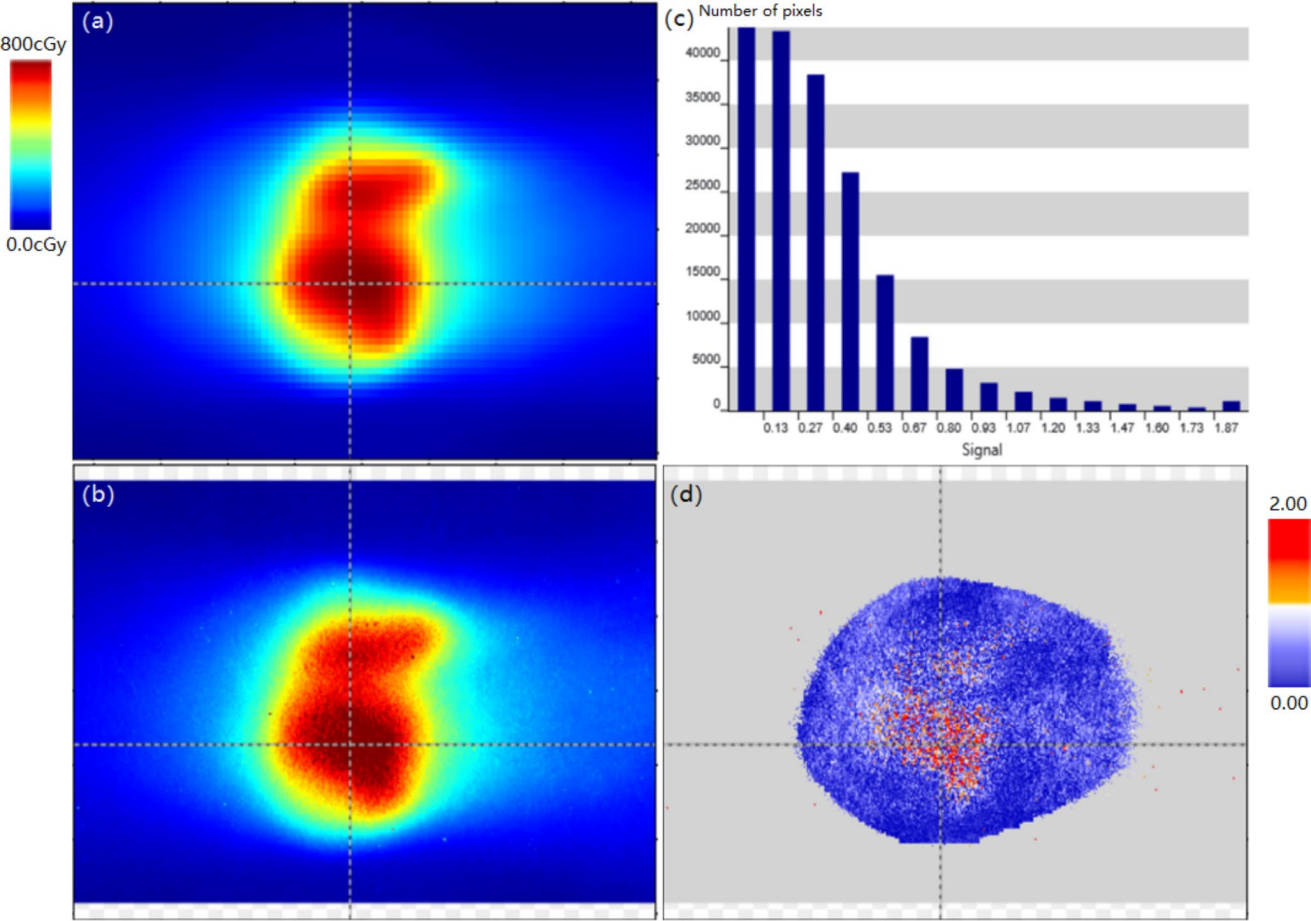
E2E test of the brain case for the focusing gamma system. **a** Plan dose distribution. **b** Measured film dose distribution. **c** Histogram of gamma index. **d** Gamma index image with 2%/1mm (10% low dose threshold)

The point dose differences of the E2E tests were − 2.57% (brain) and − 1.81% (lung), respectively. The EBT3 film measurements had gamma passing rates (2%/1 mm/10%) of 95.3% (brain) and 96.9% (lung), respectively. Figure 9 shows an example of the brain case, the gamma passing rate was 95.3% using a criterion of 2%/1 mm/10%. All the measurement results were within the tolerance levels (point dose difference ≤ 5% according to AAPM TG-21 [27] and gamma passing rate ≥ 90%).

## The kV imaging system

### A image quality

Table 7 summarizes the outcomes of the image quality. The image quality parameters fully complied with the manufacturer’s specifications regarding the CNR, spatial resolution, and uniformity.

**Table 7:** The outcomes of the image quality

Parameter		CNR		Spatial resolution	Uniformity
kV/kV(2D)		10.5(≥8.5)		24 lp/cm(≥ 10)	/
kV CBCT(3D)	HEAD	AIR	71(≥25)	8 lp/cm (≥ 7)	0.46%(≤ 5%)
		Teflon	32(≥15)		
		Acrylic	1.9(≥0.5)		
		LDPE	10(≥4.5)		
	BODY	AIR	64(≥25)	6 lp/cm (≥ 5)	1.87%(≤ 10%)
		Teflon	61(≥15)		
		Acrylic	2.2(≥0.5)		
		LDPE	13(≥4.5)		

### Geometric accuracy

**Fig. 10:**
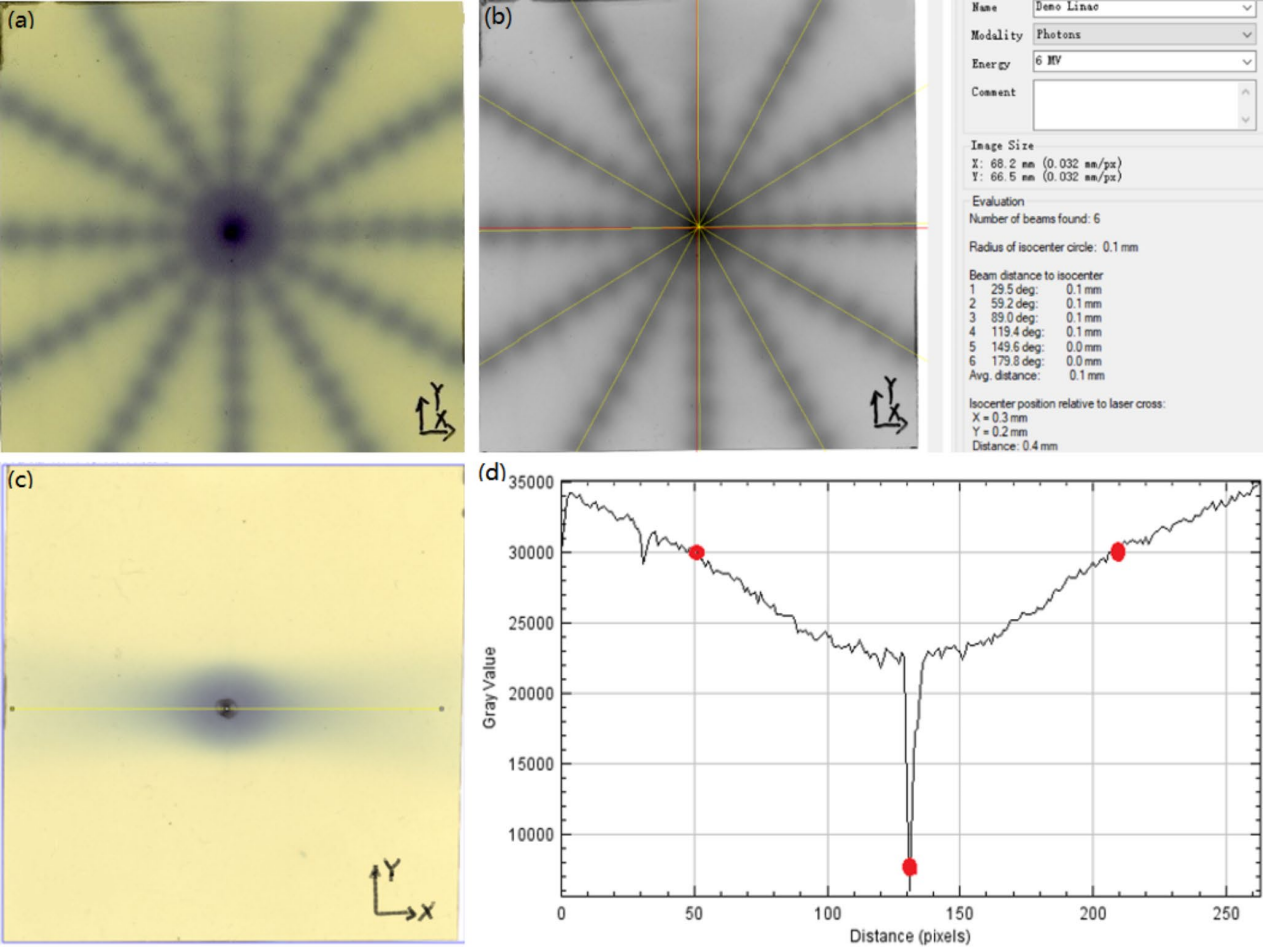
The coincidences between the imaging isocenter and linac/gamma treatment isocenter. **a** An irradiated film of star shot plans with the linac. **b** The analytical results of the coincidence between the imaging isocenter (red line) and the linac treatment isocenter (yellow line) (XOY). **c** An irradiated film of gamma plans with a 6 mm collimator shot with the focusing gamma system. Make a profile through the marker on the film. **d** The analytical results of the coincidence between the imaging isocenter and the gamma treatment isocenter (XOY). The result is the distance between the midpoint (treatment isocenter) of the two upper red points and the lower red point (imaging isocenter) using an equivalence relation (1 mm = 11.8 pixels)

The components of the image-guided treatment system are fixed on the gantry, which means that there is no flexion of the imaging and treatment components during gantry rotation so as to reduce the uncertainty of the imaging and mechanical isocenter, which shares the same isocenter with the O-ring gantry. The coincidences between the imaging isocenter and linac/gamma mechanical isocenter were both within 0.5 mm. The irradiated films and analytical results are shown in Fig. 10.

## Discussion

The MPPG 5.a provides a concise guideline to examine the TPS beam models with various geometries [13]. In this study, we have performed all applicable validation tests recommended by the MPPG 5.a, and all the test results were within the tolerance levels. However, limitations of the out-of-field dose modeling were found. For large fields (≥ 20 cm), out-of-field doses between the measured and calculated were not matched very well at deeper (≥ 20 cm) depths, the calculated doses by the TPS were lower than the measured doses. Underestimation of the out-of-field doses has been reported for both Pinnacle [28] and Eclipse [25, 29, 30]. Therefore, more attention should be paid when assessing the out-of-field doses for certain treatment situations such as testis and ovary or implantable cardiac device doses.

The TaiChi platform has received the US FDA 510k(K210921) and National Medical Products Administration (NMPA) clearance in China (20,223,050,973) currently. Since the TaiChi platform was put into clinical treatment, it exhibits good performance in mechanical, dosimetry accuracy and treatment efficiency. These clinical advantages benefit from some innovations of this platform. The first is the O-ring structure, which has the characteristics of firm structure and balanced force, and can keep the movement accuracy for a long time. The second is the capability of continuous rotation of the gantry. Based on slip ring technology, the gantry can rotate continuously clockwise and counterclockwise, which provides a more flexible selection of gantry rotation angles in treatment planning and higher treatment efficiency in some cases such as treating a posterior target using the VMAT technique. Third, unlike the Gamma Knife which utilizes 192 Cobalt-60 sources providing initial activity of 5091 Ci [31], the focusing gamma system uses 18 sources providing initial activity of 22,860 Ci with low leakage radiation of < 1 µSv/h at approximately 1 m from the location of the source and < 0.04 mSv/h at the isocenter, allowing a higher workload for the system and lower radiation dose to the radiation personnel. By rotating the Cobalt-60 γ-ray beams during treatment, 18 non-overlapping full 360° arcs are formed, resulting in high focal dose uniformity and small focal spot penumbra. The focusing gamma head can also swing 14° toward the patient’s head while keeping the treatment isocenter unchanged, giving the system more non-coplanar freedom to increase the dose gradient when treating small lesions. Finally, the linac, focusing gamma system and kV imaging system are coplanar and share the same isocenter, and the components of the system are fixed on the gantry, which means that there is less sagging for the imaging and treatment components compared to the conventional C-arm linac, and therefore improve the imaging/mechanical isocenter accuracy and reduce the blurring effects on CBCT imaging [17, 32].

The TaiChi platform integrates the linac, focusing gamma system, and kV imaging system together. It can provide x-ray intensity-modulated radiotherapy and γ-ray stereotactic radiotherapy on the same platform. The two modalities can be used for radiosurgery and radiotherapy alone, and can also be combined to produce complex dose distributions for various radiotherapy requirements. Clinically, some small target volumes can be treated much better with the focusing gamma system [33, 34] while other large, irregularly shaped target volumes are better suited for linac/MLC beams. These patients can be treated on the same platform, which is useful to therapy centers with only one treatment machine. On the other hand, multiple targets can be treated in the same treatment setting. For example, intracranial metastatic lesions can be treated with the focusing gamma system and the primary tumor in the thorax can be treated with the linac in the same treatment session, without re-setting the patient. Furthermore, the dual-treatment head system also allows the combination of γ-ray SRS/SBRT and x-ray IMRT/VMAT to optimize complex dose distributions of both modalities based on special dose fractionation schemes. Future clinical studies to explore the benefits of the multiple-modality platform are warranted.

## Conclusion

This paper summarized the technological characteristics and commissioning of a new multi-modality radiotherapy platform, the TaiChi platform. The validation results demonstrate that this platform exhibits good performance in mechanical, dosimetry accuracy and treatment efficiency and is adequate for clinical usage. Based on the multi-modality, this platform can provide optimization and personalization of radiotherapy, thereby improving the quality of radiotherapy.

## Electronic supplementary material

Below is the link to the electronic supplementary material.

Supplementary Material 1
