# Supplementary material for: Characterization and commissioning of a new collaborative multi-modality radiotherapy platform
Source: Phys Eng Sci Med. 2023 Jun 28;46(3):981–94. doi: 10.1007/s13246-023-01255-2 (PMC10480288; doi:10.1007/s13246-023-01255-2)
Supplement: Supplementary file 1 — Supplementary Material 1 [file 13246_2023_1255_MOESM1_ESM.docx]

### Supplementary Materials


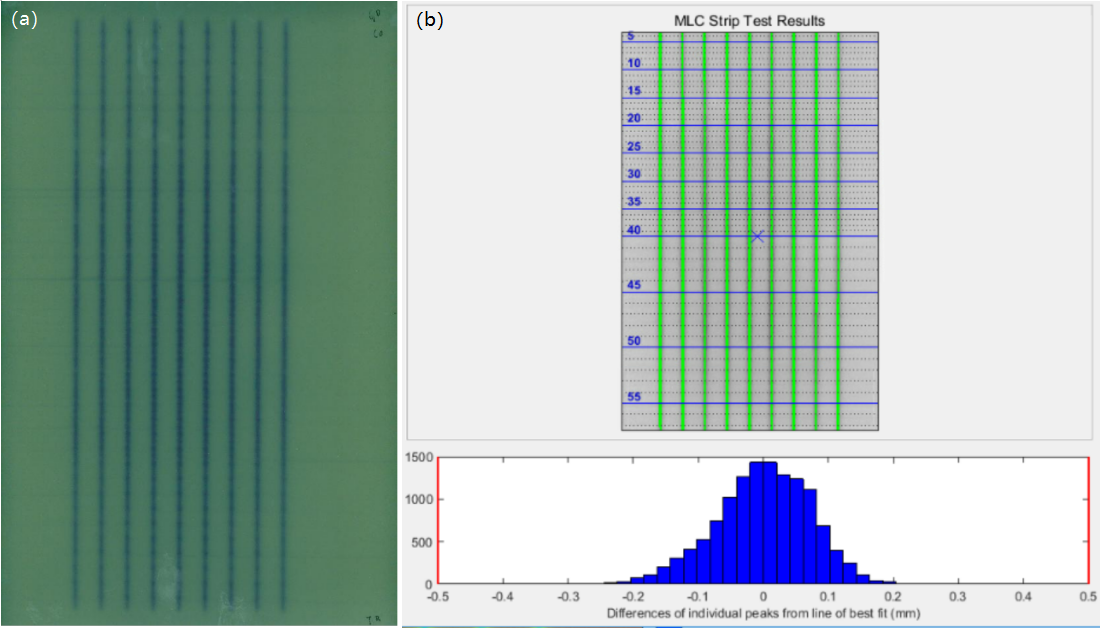


Fig. S1. An example of the result of Picket Fence test (gantry 0°, collimator 0°). (a) An irradiated film of nine strip fields with 1 mm width and 20 mm gaps. (b)The analytical results with the Doselab software.


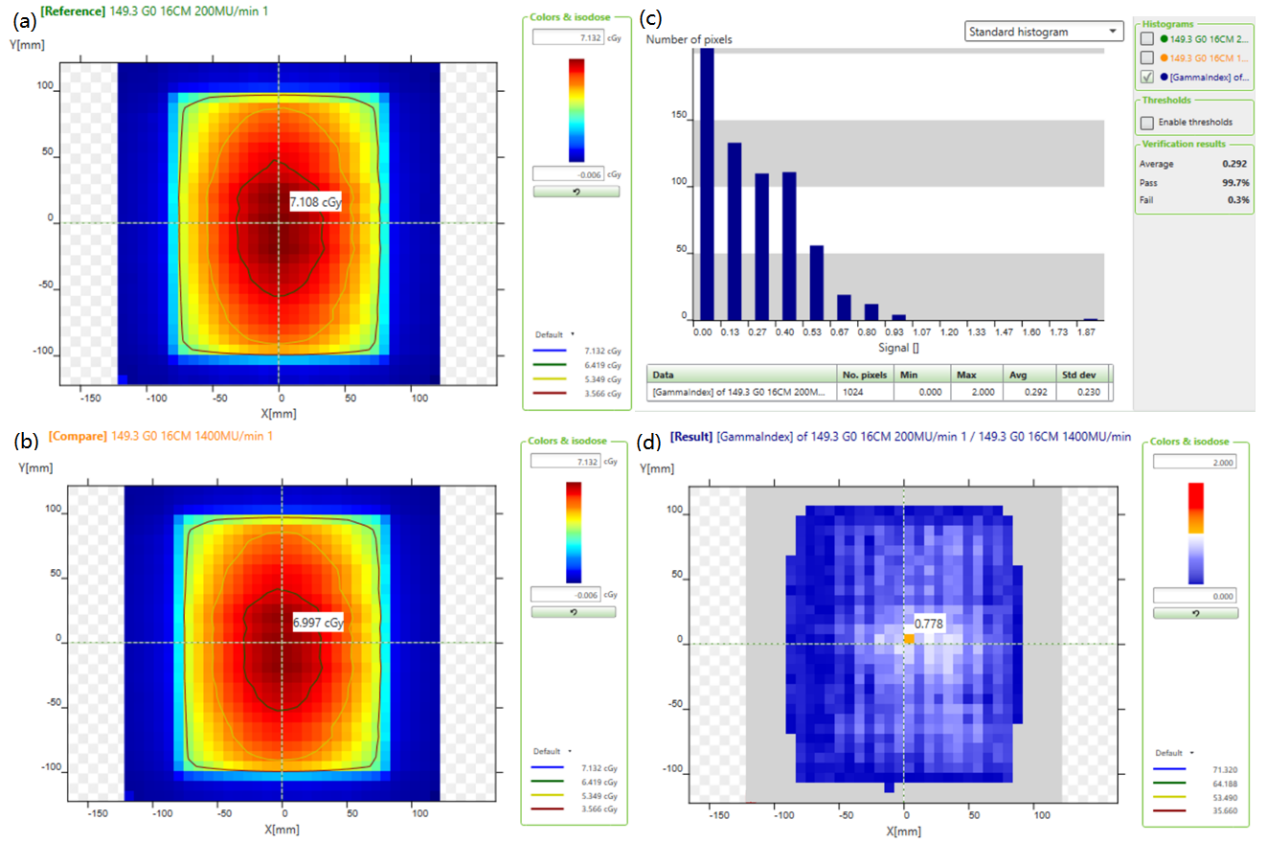


Fig. S2. An example of the result of travel speed test. Gamma passing rate of the plan delivered at 1400 MU/min vs 200 MU/min is 99.7%.(a) Dose distribution with dose rate of 200 MU/min. (b) Dose distribution with dose rate of 1400 MU/min.(c).Histogram of gamma index. (d) Gamma index image with 2%/2mm.

Table S1. An overview of the acceptance testing.

| Item | Result | Tolerance |
| --- | --- | --- |
| **Linac** | | |
| Gantry angle indicators | 0.10° | 0.5° |
| Collimator angle indicators | 0.12° | 0.5° |
| Couch position indicators | 0.6mm | 1mm |
| Couch travel maximum range  movement in all directions | X direction: 300mm  Y direction: 1860mm  Z direction: 402mm | X direction≥300mm  Y direction≥1850mm  Z direction≥400mm |
| Jaw position indicators | 0.23mm | 1mm |
| Collimator rotation isocenter | 0.3mm | 1mm |
| Gantry rotation isocenter | 0.35mm | 1mm |
| Coincidence of radiation and  mechanical isocenter | Gantry: 0.1mm  Collimator:0.1mm | 1mm |
| Depth of the maximum dose (dmax) | 13.9mm | (15±2)mm |
| Beam quality | 63.3% | (64.0±2)% |
| Penetrative quality | 139.6mm | (142±2)mm |
| Beam symmetry(three open fields) | 5×5 cm^2^  inline:1.15%; crossline:0.73%  10×10 cm^2^  inline:0.61%; crossline:1.2%  35×35 cm^2^  inline:0.67%; crossline:1.16% | 3% |
| Penumbra(three open fields) | 5×5 cm^2^: 5.6mm  10×10 cm^2^: 6.4mm  35×35 cm^2^: 8.9mm | 5×5 cm^2^:≤7mm  10×10 cm^2^:≤8mm  35×35 cm^2^:≤11mm |
| Output linearity | 0.9% | 2% |
| Output constancy | 0.15% | 0.5% |
| Output constancy vs dose rate | 0.2% | 1% |
| Output constancy vs gantry angle | 0.6% | 1% |
| **Gamma system** | | |
| Timer accuracy | 0.35s | 1s |
| Gamma swing angle accuracy | 0.2° | 1° |
| Dose rate at focus piont (Φ35 mm collimator) | 0.8% | 1.5% |
| Filed size(7 collimators) | Φ6mm:0.6mm;Φ9mm:0.7mm;  Φ12mm:0.3mm;Φ16mm:0.2mm;  Φ20mm:0.2mm;Φ25mm:-0.5mm;  Φ35mm:-0.4mm | Φ6-20mm:1mm  Φ25-35mm:2mm |
| Coincidence of radiation and mechanical isocenter | 0.37mm | 1.25mm |
| Plan point dose | Refer to 5.1. Point dose verification | 5% |
| Relative output factors | Refer to 5.2. ROF verification | 3% |
| **Imaging system** | | |
| Image quality (kV/kV, kV CBCT) | Refer to Table 7 | Refer to Table 7 |
| Coincidence of imaging isocenter and  the mechanical isocenter | Linac:0.5mm  Gamma:0.4mm | Linac:1mm  Gamma:0.5mm |
